# The combination of *Elephantopus scaber* and *Sauropus androgynus* promotes erythroid lineages and modulates follicle-stimulating hormone and luteinizing hormone levels in pregnant mice infected with *Escherichia coli*

**DOI:** 10.14202/vetworld.2021.1398-1404

**Published:** 2021-05-31

**Authors:** Muhammad Sasmito Djati, Yuyun Ika Christina, Muhaimin Rifa’i

**Affiliations:** 1 Department of Biology, Faculty of Mathematics and Natural Sciences, Brawijaya University, Malang 65145, East Java, Indonesia; 2 Doctoral Program, Department of Biology, Faculty of Mathematics and Natural Sciences, Brawijaya University, Malang 65145, East Java, Indonesia

**Keywords:** *Elephantopus scaber*, erythropoiesis, follicle-stimulating hormone, luteinizing hormone, *Sauropus androgynus*

## Abstract

**Background and Aim:**

*Escherichia coli* infection produces an adverse effect on the erythrocyte lineage and hormone levels during pregnancy. This study aimed to evaluate the effects of *Elephantopus scaber* (ES) and *Sauropus androgynus* (SA) in combination on circulating follicle-stimulating hormone (FSH) and luteinizing hormone (LH) levels and erythropoiesis changes in *E. coli*-infected pregnant mice.

**Materials and Methods:**

Female Balb/c mice were mated with normal male mice and pregnancies were identified by the formation of vaginal plugs. Twenty-eight pregnant mice were divided randomly into seven groups: A control group (N), *E. coli-*infected pregnant mice (K+), and infected pregnant mice received the following five treatments: (1) Only ES; (2) ESSA1 (75:25); (3) ESSA2 (50:50); (4) ESSA3 (25:75); and (5) only SA, beginning from the 1^st^ to the 16^th^ day of pregnancy. Pregnant mice were infected with 10^7^ CFU/mL of *E. coli* on day 4. Blood serum was collected on days 8, 12, and 16 of pregnancy and LH and FSH levels were measured by enzyme-linked immunosorbent assay. Bone marrow was isolated to determine the relative number of TER-119^+^VLA4^+^ and TER-119^+^CD34^+^ using flow cytometry.

**Results:**

The ESSA1 and SA groups exhibited a marked increase in LH levels. The combination of ES and SA administered at a 25:75 ratio (ESSA3) altered FSH levels and the relative number of TER-119^+^VLA4^+^ in infected pregnant mice. Combined with SA at an equal ratio (50:50), ESSA2 group exhibited a significant increase in the expression of TER119^+^CD34^+^ compared with the other treatment groups.

**Conclusion:**

ES and SA combined at a ratio of 25:75 exhibited optimal results in altering hormonal and erythropoiesis in infected pregnant mice.

## Introduction

Pregnancy is a unique condition characterized by several physiological alterations, including hematological and hormonal changes [1]. Erythropoietin (EPO) and erythrocyte production is increased during normal pregnancy. In contrast, erythrocyte mass per unit body weight remains constant throughout pregnancy, whereas hemoglobin and hematocrit decrease continuously into the third trimester. Erythrocyte levels are reduced during normal pregnancy because of emergency hematopoiesis in response to elevated EPO levels [2]. *Escherichia coli* is a common pathogenic bacterium that is of great concern in most countries [3]. A related study demonstrated that the innermost region of the lipopolysaccharide endotoxin of *E. coli*, also known as lipid A, increases the red blood cell (RBC) cytosolic Ca^2+^ levels and decreases RBC deformability [4]. Intracellular calcium (Ca^2+^) plays a key role in RBC physiology and survival [5]. Increased levels of Ca^2+^ cause redistribution of the RBC membrane phospholipid phosphatidylserine from the inner to the outer membrane leaflets and generate an RBC signal to promote senescence, eryptosis, and clearance [6]. Al-Zamely and Falh [7] reported that treatment with 1×10^9^ CFU/mL of *E. coli* decreases the hemoglobin concentration, caused by a breakdown of erythrocytes by hemolysis enzymes produced by *E. coli*. A decrease in erythrocytes during pregnancy results in anemia resulting from a short erythrocyte life span [8]. Bacterial infections can produce adverse effects on the erythrocyte lineage and hormone levels during pregnancy [2].

*Sauropus androgynus* (SA), or Katuk, is a medicinal plant used commonly to treat cough and fever, and as a lactation enhancer. This plant has several biological activities, including a hormonal balancing effect in infected pregnant mice [9], as well as antioxidant and antibacterial activities [10]. SA contains several vitamins, such as Vitamin C, B1, B2, B3, C, and E. This plant also has mineral content that could improve the erythropoiesis process [11]. *Elephantopus scaber* (ES), or Tapak Liman, is a medicinal plant used to treat several diseases, including hepatitis, fever, cancer, and scabies. This plant has an anti-inflammatory effect caused by the active compounds, deoxyelephantopin and isoscabertopin [12]. Djati *et al*. [13] demonstrated that the combination of ES and SA acted synergistically to balance the level of erythrocyte precursors (TER119^+^VLA4^+^), mature erythrocytes (TER119^+^VLA4^-^), and prolactin levels in pregnant mice infected with *Salmonella* Typhi or typhoid. Christina *et al*. [9] also demonstrated that combining ES and SA at a ratio of 75:25 protected renal and hepatic regions from *E. coli* infection and balanced several reproductive hormones including estrogen, progesterone, and prolactin in *E. coli*-infected pregnant mice.

A previous study focused on the effects of both plants on erythropoiesis and prolactin hormone levels following *S*. Typhi infection [13] and progesterone, estrogen, and prolactin hormone levels after *E. coli* infection [9]. However, the effects of ES and SA on erythroid progenitor (TER119^+^VLA^+^ and TER119^+^CD34^+^) changes and follicle-stimulating hormone (FSH) and luteinizing hormone (LH) levels resulting from an *E. coli* infection have not been established. Therefore, in this study, we evaluated the effect of combined ES and SA extract on circulating FSH and LH concentrations and erythropoiesis levels at the early, middle, and late stages of mouse gestation in the presence of *E. coli* infection.

## Materials and Methods

### Ethical approval

All experimental animal protocols were approved by the Animal Care and Use Committee of Brawijaya University (Approval ref no: 902-KEP-UB).

### Study period and location

The study was conducted from April to September 2020 at Laboratory of Animal Physiology, Department of Biology, Faculty of Mathematics and Natural Sciences, Brawijaya University, Malang, Indonesia.

### Extract preparation

Fresh leaves of ES and SA were collected from UPT Laboratorium Herbal Materia Medica Batu, Malang, East Java, Indonesia. The plants were identified and validated by UPT Laboratorium Herbal Materia Medica Batu. The specimen numbers (074/228/102.7/2018; 074/229/102.7/2018) were deposited into the herbarium of the Biology Department for future reference. The leaves were cleaned, dried in an oven at 40°C, and ground into powder using a laboratory blender. Powdered samples (100 g) from each plant were extracted separately by steeping in 900 mL of 95% ethanol 3 times overnight and placed in the dark. Following extraction, the samples were filtered through Whatman filter paper No. 1 (Whatman Ltd., England). The purified extracts were evaporated to dryness using a rotary evaporator at 40°C and then refrigerated at 4°C for further analysis. The crude extracts from each plant were prepared at different concentrations for animal studies.

### Animals and experimental design

Sixty-three female BALB/c mice (each weighing 20-25 g, 6 weeks old) were obtained from LPPT Gadjah Mada University, Yogyakarta, Indonesia. The mice were maintained in cages under stable conditions, which included free access to a standard diet and water, a controlled light cycle (12 h light/12 h dark), and constant temperature (22±2°C). All animals were acclimatized for 1 week before the beginning of the study.

After the acclimatization period, the mice were mated randomly by putting female and male mice into the same cage. After a vaginal plug was found, a vaginal swab was performed to determine the metestrus phase of the mice. The presence of the vaginal plug and metestrus phase in female mice was considered day 1 of the gestational period.

A total of 63 pregnant female BALB/c mice were divided randomly into seven groups (n=4) and treated as follows: Control groups with healthy pregnancies (N); pregnant mice infected with *E. coli* as a positive control (K+); pregnant mice infected with *E. coli* and treated only with 200 mg/kg ES; pregnant mice infected with *E. coli* and treated with 150 mg/kg ES and 37.5 mg/kg SA (75:25) (ESSA1); pregnant mice infected with *E. coli* and treated with 100 mg/kg ES and 75 mg/kg SA (50:50) (ESSA2); pregnant mice infected with *E. coli* and treated with 50 mg/kg ES and 112.5 mg/kg SA (25:75) (ESSA3); and pregnant mice infected with *E. coli* and treated only with 150 mg/kg SA.

The administration of ES and SA combined was initiated on days 1 through 16 of the pregnancy period. Pregnant mice were injected intraperitoneally with 10^7^ CFU/mL of *E. coli* (dissolved in 0.1 mL of phosphate-buffered saline) on day 5 of pregnancy. *E. coli* infection in pregnant mice was confirmed 24 h after injection using Gram staining and a catalase test. The results demonstrated that the infected groups had been successfully infected with *E. coli*. The pregnant mice were then treated with combined ES and SA by oral gavage daily for 16 days.

### Hormonal assessments

Mouse blood serum was collected from the orbit of the eye. LH and FSH levels were measured using an enzyme-linked immunosorbent assay (ELISA). The FSH and LH kits used in this study were the Mouse FSH ELISA kit (catalog no.: E-EL-M0511) and the Mouse LH ELISA Kit (catalog no: E-EL-M0057) (Elabscience^®^ Biotechnology Inc., USA).

### Immunostaining and flow cytometry

Bone marrow cells were isolated by flushing the femur and tibia of mice. A suspension of the cells was centrifuged at 2500 rpm at 4°C for 5 min. Cells were stained with a combination of specific antibodies: Fluorescein isothiocyanate-conjugated rat anti-mouse CD34, phycoerythrin (PE)-conjugated rat anti-mouse VLA4, and PE-CY5-conjugated rat anti-mouse TER199. The number of TER199^+^VLA4^+^ and TER199^+^CD34^+^ cells was measured by flow cytometry FACSCalibur™ (BD Biosciences, California, USA). Data were analyzed with BD cell quest Pro™ software (BD Biosciences).

### Statistical analysis

Data were analyzed using two-way analysis of variance followed by Tukey’s test. p<0.05 was considered statistically significant. All statistical analyses were performed using SPSS Statistics for Windows, Version 27.0. (IBM Corp., NY, USA) .

## Results

### FSH and LH levels

In the control group, FSH levels fluctuated from days 8 to 16 during the pregnancy period. The level of FSH in infected mice was decreased significantly compared with the control groups (p<0.05) on days 8-16 of pregnancy (Table-1). The highest level of FSH was observed in the ESSA3 group on day 8; ESSA2 and ESSA3 groups on day 12; and ESSA1, ESSA3, and SA groups on day 16 of pregnancy. These findings indicate that the ratio of combined ES and SA at 25:75 mostly caused increased FSH levels in infected pregnant mice on all the observed days of pregnancy.

**Table-1:** Effect of combined ES and SA ethanol extract on FSH levels of the experimental mice.

Group	FSH levels (ng/mL)		

	Day-8	Day-12	Day-16
N	24.55±1.73^c^	51.30±0.67^c^	29.12±2.74^c^
K+	12.33±1.71^a^	39.83±0.79^b^	19.12±0.16^b^
ES	25.62±2.68^c^	28.61±0.75^a^	11.92±1.67^a^
ESSA1	17.35±1.53^b^	26.61±1.43^a^	31.95±0.76^c^
ESSA2	20.54±0.37^b^	44.17±0.31^c^	22.12±1.84^b^
ESSA3	27.19±2.12^c^	41.13±1.00^c^	26.58±2.15^c^
SA	21.40±1.19^b^	31.57±0.69^a^	29.83±0.82^c^

Values represent mean±SD, n=4. Healthy pregnant mice were not subjected to *E. coli* infection (N), pregnant mice infected with *E. coli* (K+), pregnant mice infected with *E. coli* and treated with 200 mg/kg of ES (ES), pregnant mice infected with *E. coli* and treated with combined 150 mg/kg ES and 37.5 mg/kg SA (75% ES:25% SA), 100 mg/kg ES and 75 mg/kg SA (50% ES:50% SA), and 50 mg/kg ES and 112.5 mg/kg SA (25% ES:75% SA) (ESSA1, ESSA2, and ESSA3, respectively), and pregnant mice infected with *E. coli* and treated with 150 mg/kg SA (SA). Different superscript letters indicate statistical significance in the same column (p<0.05). ES=*Elephantopus scaber*, SA=*Sauropus androgynus,* FSH=Follicle-stimulating hormone, LH=Luteinizing hormone, *E. coli=Escherichia coli*

This study also found that LH levels in pregnant mice were decreased significantly (p<0.05) after *E. coli* infection on days 8 and 16 of pregnancy. After treatment with 50 mg/kg ES and 112.5 mg/kg SA (ESSA3 group), the LH levels increased significantly compared with the combination treatment on day 8 of pregnancy. On day 16 of pregnancy, the ESSA1 and SA groups exhibited an increase in LH levels compared with the other combination groups (Table-2).

**Table-2:** Effect of combined ES and SA extract on LH levels of infected pregnant mice.

Group	LH levels (ng/mL)		

	Day-8	Day-12	Day-16
N	122.84±3.73^c^	93.70±2.60^a^	111.98±2.16^c^
K+	112.33±2.68^b^	120.32±4.02^b^	105.56±2.48^b^
ES	84.42±1.68^a^	71.41±3.20^a^	93.10±1.25^a^
ESSA1	116.36±1.95^c^	161.75±2.97^b^	106.22±1.06^b^
ESSA2	111.84±1.08^b^	162.86±2.88^b^	99.81±1.09^a^
ESSA3	115.56±2.54^c^	202.96±2.07^c^	95.44±2.92^a^
SA	114.64±2.02^b^	217.70±5.32^c^	105.77±0.67^b^

Values were expressed as mean±SD, n=4. Healthy pregnant mice were not subjected to *E. coli* infection (N), pregnant mice infected with *E. coli* (K+), pregnant mice infected with *E. coli* and treated with 200 mg/kg of ES (ES), pregnant mice infected with *E. coli* and treated with combined 150 mg/kg ES and 37.5 mg/kg SA (75% ES:25% SA), 100 mg/kg ES and 75 mg/kg SA (50% ES:50% SA), and 50 mg/kg ES and 112.5 mg/kg SA (25% ES:75% SA) (ESSA1, ESSA2, and ESSA3, respectively), and pregnant mice infected with *E. coli* and treated with 150 mg/kg SA (SA). Different superscript letters indicate statistical significance in the same column (p<0.05). ES=*Elephantopus scaber,* SA=*Sauropus androgynus,* FSH=Follicle-stimulating hormone, LH=Luteinizing hormone, *E. coli=Escherichia coli*

### Effect of combined ES and SA on TER-119^+^VLA4^+^ cell expression

**Figure-1:**
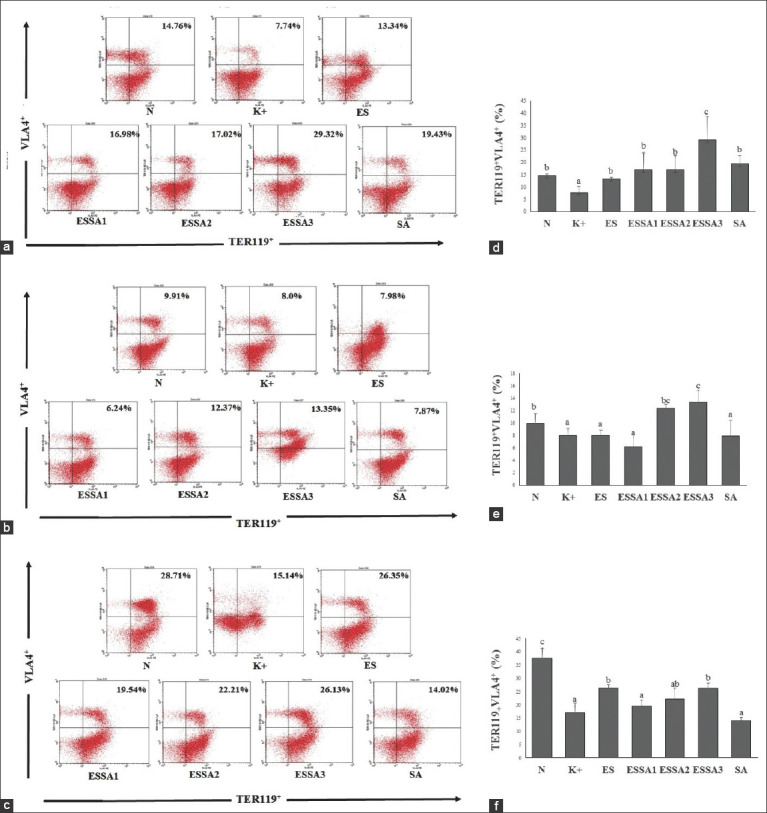
Flow cytometry of isolated bone marrow labeled with TER-119^+^VLA^+^ post-*Escherichia coli* challenge. (a-c) Representative FACS dot plots showing the percentage of TER-119^+^VLA^+^ in all groups treatment on day-8, 12, and 16, respectively. (d-f) The bars are a calculation of the relative number TER-119^+^VLA^+^ expression on day-8, 12, and 16, respectively. The results were expressed as the mean±SD. Different superscript letters indicate statistical significance (p<0.05). Note: Healthy pregnant mice were not subjected to *E. coli* infection (**N**), pregnant mice infected with *E. coli* (**K+**), pregnant mice infected with *E. coli* and treated with 200 mg/kg of ES (**ES**), pregnant mice infected with *E. coli* and treated with combined 150 mg/kg ES and 37.5 mg/kg SA (75% ES:25% SA), 100 mg/kg ES and 75 mg/kg SA (50% ES:50% SA), and 50 mg/kg ES and 112.5 mg/kg SA (25% ES:75% SA) (**ESSA1, ESSA2,** and **ESSA3,** respectively), and pregnant mice infected with *E. coli* and treated with 150 mg/kg SA (**SA**).

Based on flow cytometry, we found that the relative number of TER-119^+^VLA4^+^ cells was decreased significantly in *E. coli-*infected pregnant mice on days 8–16 of the pregnancy period (Figure-1). Interestingly, treatment with 50 mg/kg ES and 112.5 mg/kg SA (ESSA3 group) resulted in a marked increase in TER-119^+^VLA4^+^ compared with the other treatment groups on days 8 and 16 of the pregnancy period. Furthermore, the ES group also contained a relatively high number of TER-119^+^VLA4^+^ cells on day 16 (Figures-1c and 1f). These findings indicate that ES alone and in combination with SA significantly increase the relative number of TER-119^+^VLA4^+^ cells in infected pregnant mice.

### Effect of combined ES and SA on TER119^+^CD34^+^ cell expression

**Figure-2:**
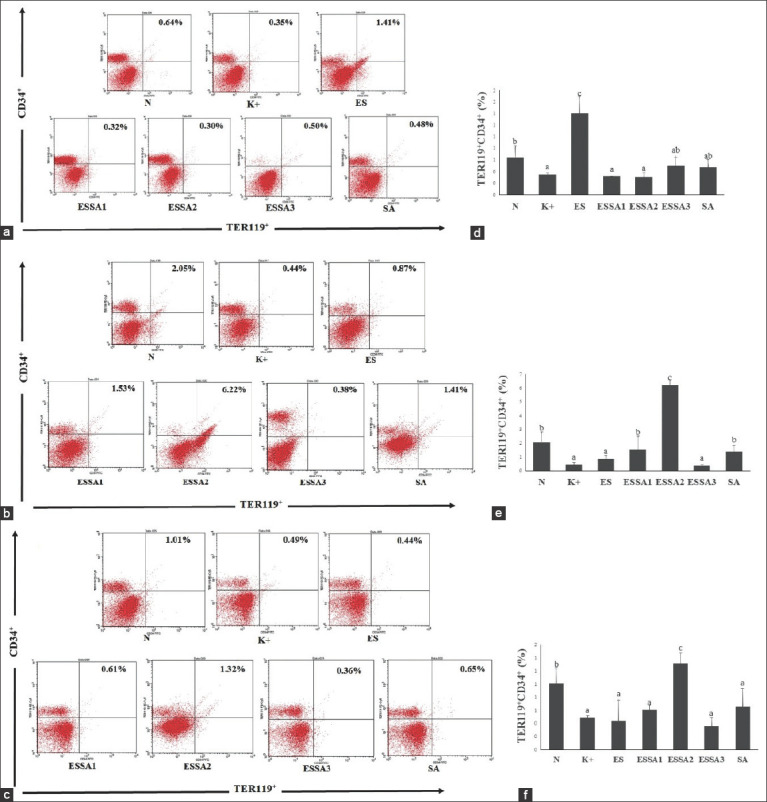
Flow cytometry of isolated bone marrow labeled with TER-119^+^CD34^+^ post-Escherichia coli challenge. (a-c) Representative FACS dot plots showing the percentage of TER-119^+^CD34^+^ in all groups treatment on day-8, 12, and 16, respectively. (d-f) The bars are a calculation of the relative number TER-119^+^CD34^+^ expression on day-8, 12, and 16, respectively. The results were expressed as the mean±SD. Different superscript letters indicate statistical significance (p<0.05). Note: Healthy pregnant mice were not subjected to *E. coli* infection (N), pregnant mice infected with *E. coli* (K+), pregnant mice infected with *E. coli* and treated with 200 mg/kg of ES (ES), pregnant mice infected with *E. coli* and treated with combined 150 mg/kg ES and 37.5 mg/kg SA (75% ES:25% SA), 100 mg/kg ES and 75 mg/kg SA (50% ES:50% SA), and 50 mg/kg ES and 112.5 mg/kg SA (25% ES:75% SA) (ESSA1, ESSA2, and ESSA3, respectively), and pregnant mice infected with *E. coli* and treated with 150 mg/kg SA (SA).

We found that the expression of TER119^+^CD34^+^ cells in the bone marrow of pregnant mice was decreased significantly after infection with *E. coli* compared with healthy pregnant mice. On day 8, the expression of TER119^+^CD34^+^ in infected pregnant mice tended to increase after treatment with 200 mg/kg ES extract (ES group) (Figures-2a and 2d). Interestingly, on days 8 and 16, the ESSA2 group (50% ES:50% SA) exhibited a significant increase in the expression of TER119^+^CD34^+^ compared with the other treatment groups (Figure-2). These findings indicate that an equal ratio of ES and SA significantly enhances the expression of erythroid progenitors expressing CD34 in TER119 cells.

## Discussion

This study demonstrated a significant rise in plasma LH levels during early pregnancy (day 8) in mice post-implantation. According to Sengupta [14], LH peaks during normal mouse pregnancy can be observed on days 4 and 11 of gestation, without significant changes in FSH. The sharp increases in FSH and LH were found only on the day of parturition. Next, we observed that LH levels tended to decrease at mid-pregnancy (day 12) and increase again during the late pregnancy period (day 16). The increased levels of LH in various stages of pregnancy are important to maintain pregnancy and prevent spontaneous abortion. Early pregnancy loss is caused by low LH levels [15]. The low level of LH is correlated with poorer oocyte maturation and endometrium quality, which results in increased spontaneous abortions. This supports our findings that spontaneous abortion occurs in infected pregnant mice (data not shown).

The increasing LH levels are associated with progesterone production and FSH is also involved in the production of estrogen required for implantation. This study revealed that FSH levels in infected pregnant mice were increased significantly after receiving combined ES and SA at a ratio of 25:75 (Table-1). However, in a previous study [9], estrogen levels were stable after receiving *E. coli* injection along with ES and SA in combination. Several factors may explain these differences in estrogen levels after *E. coli* infection. For mice, the estrogen surge occurs on day 4 of pregnancy. After day 4 of pregnancy, the estrogen levels decrease [16]. On the 8^th^ day of pregnancy, estrogen levels were more stable than those of the controls because the estrogen surge was reduced and lower concentrations were required during this particular observation time.

Christina *et al*. [9] demonstrated that *E. coli* infection increases fetal reabsorption and decreases serum progesterone levels, which are needed during a normal mouse pregnancy. Their study suggested that hormonal changes during pregnancy produced an adverse effect [9]. The use of a powerful natural bioactive compound from plants is urgently needed to prevent the adverse effects of *E. coli* infection during pregnancy. Christina *et al*. [9] demonstrated that the ES and SA combination plays a role in balancing the prolactin and progesterone changes that occur during *E. coli* infection in pregnant mice. The effective dose of combined ES and SA that effectively alter these hormones is a ratio of 75:25, or approximately 150 mg/kg ES and 37.5 mg/kg SA [9]. Our study demonstrated that all combinations impacted hormone levels, and the most remarkable improvement in FSH and LH levels were observed in the ESSA3 group, which consisted of 25% ES and 75% SA. Katuk (SA) leaves have a significant effect on eicosanoid biosynthesis and are involved in the reproduction process and milk production in lactating sheep [17]. Several bioactive compounds of SA, such as 17-ketosteroid, androstane-17-one, and 3-ethyl-3-hydroxy-5alpha, promote female steroid hormone synthesis [18].

TER119 is a specific marker expressed at all stages of erythroid development (from early pro-erythroblasts to mature erythrocytes), whereas VLA-4 is found mostly on hematopoietic stem and progenitor cells. TER-119^+^VLA-4^+^-positive cells in the bone marrow represent an erythroid-specific marker at the early progenitor stage (pro-erythroblasts) [19]. To further evaluate the effect of bacterial infection on erythropoiesis, we isolated bone marrow cells and labeled them with TER-119 and VLA-4 antibodies. The results indicated that TER-119^+^VLA4^+^ was significantly downregulated after *E. coli* infection in pregnant mice compared with that in healthy pregnant mice. This indicated a depletion in the erythroid component of the bone marrow. A recent study demonstrated that erythropoiesis is altered significantly during bacterial infection and increases splenic erythropoiesis [20]. Our data showed that the primary effect of *E. coli* on erythroid cell development was found at all stages of mouse pregnancy.

The marked production of erythrocytes resulting in accelerated erythropoiesis supports pregnancy [21]. An accelerated production of the erythroid progenitor (TER11^9^+VLA-4+) from days 8 to 16 with the relative number of 14.76% and 37.53%, respectively. The high expression of erythroid progenitors is essential to support pregnancy because a massive decrease in erythroid progenitors causes fetal abortion in pregnant mice (data not shown). The use of ES alone or in combination with SA enhances the relative number of TER-119^+^VLA4^+^ cells in infected pregnant mice. Cells expressing CD34 are found in the umbilical cord and bone marrow as markers of progenitor cells. The presence of CD34 on TER119 cells has been linked to erythroid progenitors [22]. The expression of cells with positive TER119 and VLA-4 was decreased markedly in pregnant mice after *E. coli* infection, which was observed on days 8, 12, and 16 of the pregnancy period. The 50:50 ratio of ES and SA could increase the expression of erythroid progenitor cells markedly by TER119^+^CD34^+^.

Djati *et al*. [23] revealed that ES leaf extracts could stimulate the proliferation of the erythroid lineage (TER119^+^VLA-4^+^) in the bone marrow. The increasing level of TER119^+^VLA-4^+^ by ES alone results from a high iron (Fe) content in the ethanol extract of ES. The Fe content plays a crucial role in mouse erythropoiesis and contributes to hemoglobin development during erythropoiesis [24]. Hemoglobin is urgently needed during the erythroid stage from basophilic erythroblasts to the reticulocyte stage. However, the development of burst-forming, unit-erythroid to basophilic erythroblasts depends on EPO [25]. Flavonoid compounds may induce EPO hormone expression and contribute to the early erythropoiesis stage of erythroid progenitors [26]. Therefore, ES and SA combined at a proper ratio may prevent the damage caused by *E. coli* infection during pregnancy.

## Conclusion

This study suggests that ES and SA combined at a ratio of 25:75 exhibit optimal results to alter hormone levels and erythropoiesis during bacterial infection in pregnant mice.

## Authors’ Contributions

MSD: Conceptualized and designed the study and did the data analysis. YIC: Carried out the experiment and wrote the manuscript. MR: Reviewed and edited the manuscript. All authors read and approved the final manuscript.

## Publisher’s Notes

Veterinary World remains neutral with regard to jurisdictional claims in published institutional affiliation.

## References

[1] Taj N, Muhammad A, Mir A, Khan M.J (2019). Changes in hematological parameters during different trimesters of pregnancy. Bull. Environ. Pharmacol. Life Sci.

[2] Vega-Sánchez R, Tolentino-Dolores M.C, Cerezo-Rodriguez B, Chehaibar-Besil G, Flores-Quijano M.E (2020). Erythropoiesis and red cell indices undergo adjustments during pregnancy in response to maternal body size but not inflammation. Nutrients.

[3] Abebe E, Gugsa G, Ahmed M (2020). *Escherichia coli* O157:H7 an emerging pathogen in foods of animal origin. J. Trop. Med.

[4] Bernhardt I, Nguyen D.B, Wesseling M.C, Kaestner L (2020). Intracellular Ca^2+^ concentration and phosphatidylserine exposure in healthy human erythrocytes in dependence on *in vivo* cell age. Front. Physiol.

[5] Bateman R.M, Sharpe M.D, Singer M, Ellis C.G (2017). The effect of sepsis on the erythrocyte. Int. J. Mol. Sci.

[6] Repsold L, Joubert A.M (2018). Eryptosis:An erythrocyte's suicidal type of cell death. Biomed. Res. Int.

[7] Al-Zamely H, Falh S (2011). The effect of experimental *Escherichia coli* infection on some blood parameters and histological changes in male rats. Iraq. J. Vet. Med.

[8] de Freitas M.A.R, da Costa A.V, Medeiros L.A, Cunha L.M, Filho U.C, da Silva Garrote Filho M, Diniz A.L.D, Penha-Silva N (2019). The role of the erythrocyte in the outcome of pregnancy with preeclampsia. PLoS One.

[9] Christina Y.I, Diana M.R, Fuzianingsih E.N, Nurhayati N, Ridwan F.N, Widodo W, Rifa'I M, Djati M.S (2020). Hormone-balancing and protective effect of combined extract of *Sauropus androgynus* and *Elephantopus scaber* against *E. coli*-induced renal and hepatic necrosis in pregnant mice. J. Ayurveda Integr. Med.

[10] Wei L.S, Wee W, Siong J.Y.F, Syamsumir D.F (2011). Characterization of antimicrobial, antioxidant, anticancer properties and chemical composition of *Sauropus androgynous* stem extract. Acta Med. Lit.

[11] Petrus A.J.A (2013). *Sauropus androgynus* (L.) Merrill-a potentially nutritive functional leafy-vegetable. Asian J. Chem.

[12] Gayathramma K, Pavani K.V, Raji R (2012). Chemical constituents and antimicrobial activities of certain plant parts of *Sauropus androgynus* L. Int. J. Pharm. Bio Sci.

[13] Djati M.S, Rahma Y.A, Dwijayanti D.R, Rifai M, Rahayu S (2017). Synergistic effect of *Elephantopus scaber* L and *Sauropus androgynus* L. Merr extracts in modulating prolactin hormone and erythropoiesis in pregnant typhoid mice. Trop. J. Pharm. Res.

[14] Sengupta P (2012). Challenge of infertility:How protective the yoga therapy is?. Anc. Sci. Life.

[15] Chen C.D, Chiang Y.T, Yang P.K, Chen M.J, Chang C.H, Yang Y.S, Chen S.U (2016). Frequency of low serum LH is associated with increased early pregnancy loss in IVF/ICSI cycles. Reprod. Biomed. Online.

[16] Hirota Y, Kanzaki H (2016). Uterine receptivity in mouse embryo implantation. Uterine Endometrial Function.

[17] Fikri F, Purnama M.T.E (2020). Pharmacology and phytochemistry overview on *Sauropus androgynous*. Syst. Rev. Pharm.

[18] Djati M.S, Dwijayanti D.R, Rifa'I M (2016). Herbal supplement formula of *Elephantopus scaber* and *Sauropus androgynus* promotes IL-2 cytokine production of CD4+T cells in pregnant mice with typhoid fever. Open Life Sci.

[19] Koulnis M, Pop R, Porpiglia E, Shearstone J.R, Hidalgo D, Socolovsky M (2011). Identification and analysis of mouse erythroid progenitors using the CD71/TER119 flow-cytometric assay. J. Vis. Exp.

[20] Hyland L, Villarreal-Ramos B, Clarke B, Baaten B, Hou S (2005). Bone marrow immunosuppression in *Salmonella*-infected mice is prolonged following influenza virus infection. Exp. Hematol.

[21] Ifeanyi O.E, Uzoma O.G (2018). A review on erythropoietin in pregnancy. J. Gynecol. Womens Health.

[22] Sidney L.E, Branch M.J, Dunphy S.E, Dua H.S, Hopkinson A (2014). Concise review:Evidence for CD34 as a common marker for diverse progenitors. Stem Cells.

[23] Djati M.S, Habibu H, Jatiatmaja N.A, Rifai M (2015). Tapak liman (*Elephantopus scabe*r L) extract induced CD4+and CD8+differentiation from hematopoietic stem cell/progenitor cell proliferation of mice (*Mus musculus*). J. Exp. Life Sci.

[24] Katsarou A, Pantopoulos K (2020). Basics and principles of cellular and systemic iron homeostasis. Mol. Aspects Med.

[25] Koury M.J, Ponka P (2004). New insights into erythropoiesis:The roles of folate, vitamin B12, and iron. Annu. Rev. Nutr.

[26] Zheng K.Y, Choi R.C, Cheung A.W, Guo A.J, Bi C.W, Zhu K.Y, Fu Q, Du Y, Zhang W.L, Zhan J.Y, Duan R, Lau D.T, Dong T.T, Tsim K.W (2011). Flavonoids from *Radix astragali* induce the expression of erythropoietin in cultured cells:A signaling mediated via the accumulation of hypoxia-inducible factor-1a. J. Agric. Food Chem.

